# Resveratrol inhibits ferroptosis in the lung tissues of heat stroke-induced rats via the Nrf2 pathway

**DOI:** 10.1186/s40360-024-00810-1

**Published:** 2024-11-19

**Authors:** Liwen Du, Xueqi Zhu, Zhenluo Jiang, Weidong Wang, Peng Liu, Leilei Zhu, Fangqi Zhang

**Affiliations:** 1 Department of Emergency, Ningbo No.2 Hospital. No. 41, Northwest Street, Haishu District, Ningbo, 315010 China; 2 Department of Pulmonary and Critical Care Medicine, The 987th Hospital of Joint Logistics Support Force of Chinese PLA, Baoji, 721000 China

**Keywords:** Heat stroke, Lung injury, Ferroptosis, Resveratrol, Nrf2

## Abstract

**Background:**

Heat stroke (HS) can lead to the development of pulmonary ferroptosis. The inhibition of pulmonary ferroptosis during HS improves patient prognosis. The aim of this study was to investigate the effects of resveratrol (RES) on heat stress at an ambient temperature of 42 °C.

**Methods:**

Heat stress was induced in Beas-2B cells and lung injury was induced in HS rats at an ambient temperature of 42 °C. The anti-oxidative stress and anti-ferroptotic effects of RES were confirmed through tail vein injection of nuclear factor-2 associated factor (Nrf2) shRNA recombinant adeno-associated virus 6 (AAV6-shNrf2).

**Results:**

RES treatment attenuated the upregulation of reactive oxygen species (ROS) and malondialdehyde (MDA) levels and alleviated glutathione inhibition in HS. In addition, RES treatment reduced the accumulation of Fe^2+^ in heat-stressed Beas-2B cells and increased the ferroptosis resistance-related proteins FTH1, GPX4, and SLC7A11 as well as the anti-oxidative stress pathway proteins Nrf2, NQO1, and HO-1. The antioxidant and anti-ferroptotic effects of RES in heat-stressed Beas-2B cells were effectively reversed upon treatment with Nrf2-IN-1, an Nrf2 pathway inhibitor. In the HS rat model, the antioxidant and anti-ferroptotic effects of RES were reversed by an ambient temperature of 42 °C and relative humidity of 60 ± 5%.

**Conclusions:**

RES effectively protected HS rats from lung injury, inhibited the accumulation of Fe^2+^, ROS, and MDA in the lung, and upregulated FTH1, GPX4, SLC7A11, Nrf2, NQO1, and HO-1.

**Graphical Abstract:**

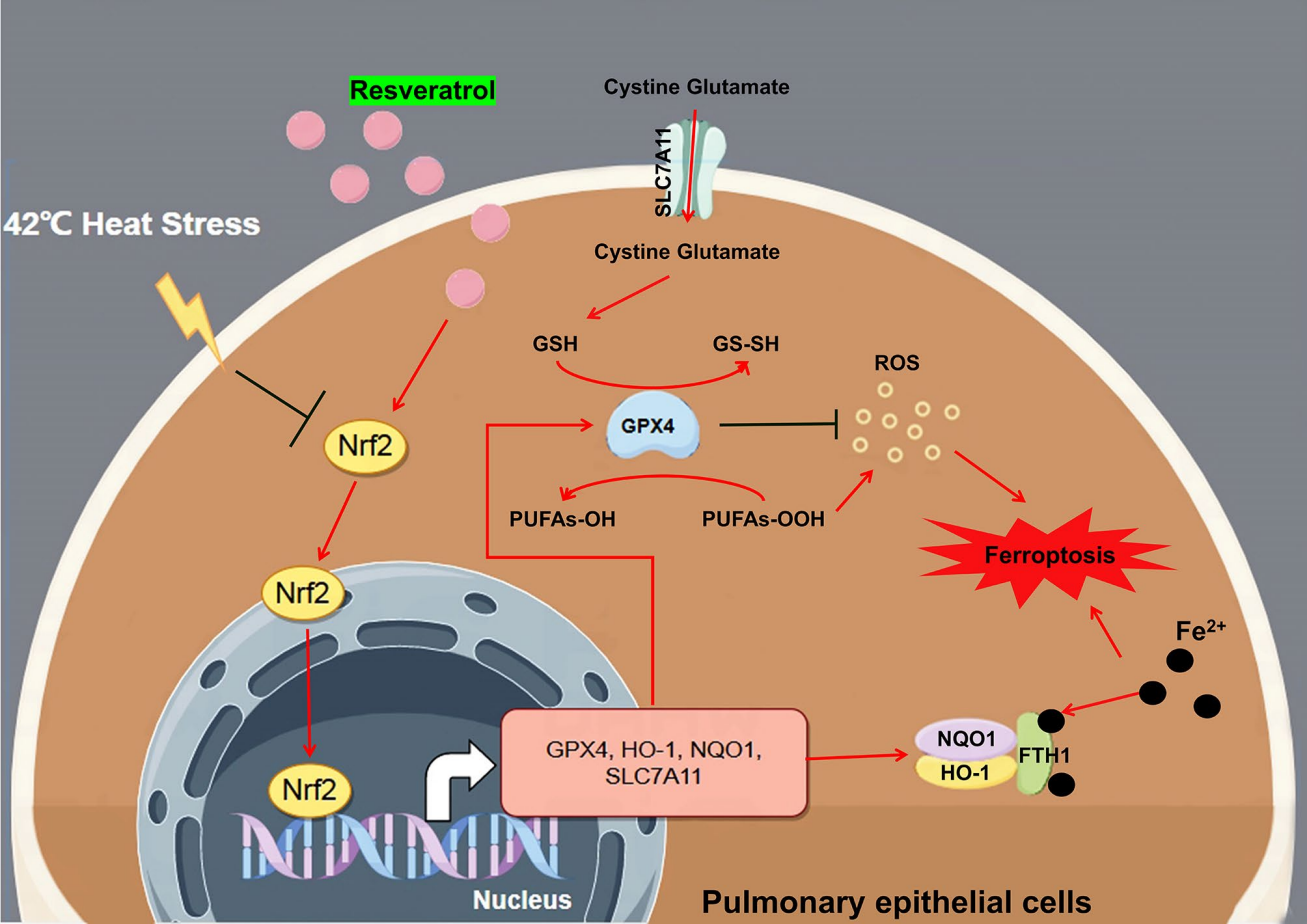

**Supplementary Information:**

The online version contains supplementary material available at 10.1186/s40360-024-00810-1.

## Introduction

Heat stroke (HS) is a somatic disorder characterized by elevated core body temperature (> 40 °C), sensory fever, central nervous system abnormalities (coma, syncope, and delirium), and multi-organ dysfunction syndrome [1]. Sustained elevation of the core body temperature may lead to tissue and organ damage and may be life-threatening in severe cases. Owing to their extensive network of capillaries, the lungs are essential organs for heat dissipation and oxygen exchange. Impaired lung function due to HS can induce and promote damage to vital tissues and organs [2].

In addition to increasing reactive oxygen species (ROS) and endogenous malondialdehyde (MDA) levels, which lead to lung injury [3–5], in recent years, some researchers have reported that heat stress can mediate lung tissue injury by regulating ferroptosis [6]. Ferroptosis is a novel form of cell death regulation, characterized by iron-dependent lipid peroxide accumulation [7]. Chen et al. reported that the heat stress-induced acute lung injury was associated with lung epithelial cell ferroptosis and that ferroptosis inhibitors could alleviate lung injury [6]. Therefore, protecting the lungs before and after heat exposure could be beneficial for improving the prognosis of patients with HS.

Resveratrol (RES) is a natural polyphenolic compound found in abundance in common fruits (such as red grapes) and vegetables and is commonly used as a health food [8]. In vivo studies have confirmed that RES can protect against acute lung injury in rats with sepsis by upregulating antioxidant cytokines such as heme oxygenase-1 (HO-1) and nuclear factor-2 associated factor (Nrf2) [9, 10]. In terms of heat stress protection, RES has been confirmed to protect the body from the effects of heat stress by upregulating heat shock-related proteins and enhancing antioxidant activity [11–13]. Interestingly, RES protects human tracheal lung epithelial Beas-2B cells from ferroptosis induced by a ferroptosis inducer, erastin, via Nrf2 signaling [14].

Based on the cytoprotective effects of RES in lung injury and heat stress, we speculated that RES might protect against the occurrence of acute lung injury caused by thermal radiation by regulating the level of oxidative stress and protecting against ferroptosis. Accordingly, here, we aimed to verify the effect of RES on the resistance to ferroptosis in lung tissue under heat stress using human lung epithelial Beas-2B cells and a heat shock rat model. This study will provide more experimental evidence for the role of RES in heat shock prevention and treatment and open new avenues for its clinical application.

## Materials and methods

### Cell culture

Human bronchopulmonary epithelial Beas-2B cells were purchased from Zhejiang Ruyao Biotechnology Co. Ltd (Ningbo, China). Cells were maintained in DMEM medium containing 10% fetal bovine serum (Gibco, USA) and 1% penicillin/streptomycin (Hyclone, USA) and placed in a cell thermostat incubator containing 5% CO_2_ at 37 °C for passaged culture.

### Effect of heat stress on cell viability

Cells were inoculated into 96-well plates at 5 × 10^3^ cells/well, and heat stress was induced after complete wall attachment. Heat stress treatment: the cells were placed in a water bath at 37 °C, 38 °C, 40 °C, and 42 °C for 2 h and then transferred to a constant temperature incubator at 37 °C for 24 h. After 24 h of culture, the cells were observed under a phase contrast microscope (DMIL, Leica, Germany). The MTT reagent (10 µL) was added to the 96-well plate, the plate was then incubated for 4 h, following which 100 µL DMSO was added. Cell viability was detected using an enzyme standard (CMax Plus, Molecular Devices, USA) to perform a cell viability assay at 560 nm.

### RES cytotoxicity study

Cells were inoculated in 96-well plates at a density of 5 × 10^3^ cells/well. After complete wall attachment, the cells were treated with final concentrations of 0, 0.02, 0.1, 0.5, 2.5, and 12.5 µM RES (501-36-0, 99.94% purity, purchased from MedChemExpress) for 24 h. The MTT reagent was then added, and cells were incubated for another 4 h. The MTT assay was performed as described above.

### Effect of RES treatment on cell viability under heat stress

Cells were inoculated in 96-well plates at a density of 5 × 10^3^ cells/well, after complete apposition, the cells were treated with 0, 0.02, 0.1, 0.5, 2.5, and 12.5 µM concentrations of RES. After the addition of RES, the cells were placed in an incubator at 42 °C for 2 h of heat stress treatment. Beas-2B cells placed in an incubator at 37 °C for 2 h were used as the control group. After the end of heat stress, the cells were transferred to a 37 °C CO_2_ (5%) incubator and cultured for another 24 h, followed by the addition of MTT reagent and detection of cell proliferation.

### Oxidative stress indicator test

Cells were seeded at a density of 1 × 10^6^ cells/well in 6-well plates for applanation culture. Cells were grouped into the following five groups: Group 1: 37 °C group, Beas-2B cells were incubated at 37 °C for 2 h. Group 2: 42 °C group, Beas-2B cells were incubated at 42 °C for 2 h. Groups 3–5: Beas-2B cells were treated with 20,100, and 500 nM RES and incubated at 42 °C for 2 h. After the above-mentioned treatments, the cells were transferred to a 37 °C incubator for 24 h. The cells subjected to the above-mentioned treatments were collected and assayed for the oxidative stress indicators MDA (ab118970), glutathione (GSH) (ab239727), and ROS (DCFH-Da probe assay, ab113851) using the respective kits (Abcam, Cambridge, UK) according to the manufacturer’s instructions. Supernatants from rat lung tissue homogenates were used to detect MDA, glutathione peroxidase (GSH-Px) (A005-1-2, Nanjing Jiancheng Institute of Biological Engineering, China), and SOD levels (A001-3-2, Nanjing Jiancheng Institute of Biological Engineering).

### LDH assay

Cell culture or tissue homogenate supernatant was collected for the lactate dehydrogenase (LDH) assay. An LDH Assay Kit (ab102526, Abcam) was used. The results were calculated by measuring the absorbance at OD_450 nm_ using an enzyme marker.

### Immunofluorescence (IF)

Cells (1 × 10^6^ cells/well) were inoculated on coverslips in 6-well plates for applanation culture. The cells were processed according to cell grouping. IF staining was performed after 24 h. Briefly, coverslips with adherent cells were fixed in 4% paraformaldehyde for about 20 min and permeabilized with 0.2% Triton X-100 for 5 min. After blocking with 1% BSA, the cells were first stained with primary antibodies (rabbit anti-GPX4 [ET1706-45, Huabio, China], SLC7A11 [ARG57998, Arigo, China], HO-1 [ab68477, Abcam], and Nrf2 [A11159, ABclona, Chinal]) and then with a secondary antibody (goat anti-rabbit IgG-FITC [ab6717, Abcam]) for 1.5 h at 25 °C under dark conditions. Afterwards, the cells were stained with DAPI for 15 min. The cells were observed and photographed using a fluorescence microscope (DM500; Leica) in three different perspectives. The results were statistically quantified using IPP6.0.

### Western blotting

Grouped treated cells (5 × 10^6^) were taken, and 100 µL of 10 × RIPA lysis buffer was added. The tissues were homogenized using a tissue crusher (pre-cooled) and then heated at 100 °C for 12 min to denature the proteins. After separation using SDS-PAGE, the proteins were transferred onto polyvinylidene fluoride membranes (Millipore, Germany). The membranes were incubated with appropriate primary antibodies (anti-human TFR1 [ab269513, Abcam], FTH1 [ET1610-78, Huabio], SLC7A11, GPX4, NQO1 [ET1702-50, Huabio], HO-1, Nrf2, and β-actin (1:1000) for 16 h at 4 °C and then with goat anti-rabbit (ab6721, 1:2000) or mouse (ab6789, 1:2000) IgG-HRP secondary antibodies at room temperature for 1 h. Chemiluminescent agents were used for visualizing the bands. A ChemiDoc-It Imaging System (UVP, USA) was used for the imaging. Results were quantified using the ImageJ software.

### Molecular simulation docking

First, the 3D structure of Nrf2 (UniProt ID: Q16236) was obtained from the AlphaFold Protein Structure Database and saved in the PDB format. The PlayMolecule platform (https://open.playmolecule.org/landing) was used to predict pocket Nrf2 activity sites. The structure of RES was obtained from the PubChem database (https://pubchem.ncbi.nlm.nih.gov/). The active pocket binding energies of RES and Nrf2 were calculated using AutoDockTools-1.5.6 according to the value of the active site. Finally, simulation docking of the most active binding site between RES and Nrf2 was visualized using the PyMOL 2.5 software.

### Effect of Nrf2 inhibition on the RES-mediated protection against heat stress

Cell grouping II: (1) 37 °C group; (2) 42 °C group; (3) 42 °C + RES group—cells were treated with 500 nM RES followed by incubation in a 42 °C water bath for 2 h; (4) 37 °C + Nrf2-IN-1 group—cells were treated with 10 µM Nrf2 inhibitor Nrf2-IN-1 (Cas no. 1610022-76-8, purity 99.89%, MCE Inc.), followed by incubation in a water bath at 37 °C for 2 h; (5) 42 °C + RES + Nrf2-in-1 group—cells were treated with 500 nM RES and 10 µM Nrf2 inhibitor (Nrf2-IN-1) and incubated in a 42 °C water bath for 2 h. The five groups of cells were uniformly transferred to a constant-temperature incubator at 37 °C at the end of water-bath incubation and incubated for another 24 h.

The MTT assay was performed at the end of incubation in a constant temperature incubator to detect the level of cell viability. The supernatant was collected to detect LDH activity. Cells (5 × 10^5^) were collected to detect the intracellular Fe^2+^ content using the Fe^2+^ Content Assay Kit (#K390-100; BioVision, Inc., USA). After obtaining 2 × 10^6^ cells for protein extraction, western blotting was performed to detect the protein expression levels of FTH1, SLC7A11, GPX4, NQO1, HO-1, and Nrf2.

### Grouping and preparation of rat HS model

A closed group of 50 male Wistar rats, weighing 300 g and approximately 12 weeks old, was selected for the experiment (Shanghai JieSiJie Laboratory Animal Co., Ltd.). The rats involved in the experiments were housed in a temperature-controlled 24 ± 2 °C rearing box with 35 ± 5% humidity. Ethical approval for the animal experiments was provided by the Experimental Animal Welfare and Ethics Committee of the Ningbo Institute of Life and Health Industry, University of the Chinese Academy of Sciences (No. GK-2023-XM-0091).

On the day of the experiment, the rats were placed in a room at a temperature of 28 °C for 30 min to rest, and the electric thermostatic incubator (chamber liner size: 25 cm × 25 cm × 25 cm) was preheated to 42 °C. The electric fan at the top of the chamber produced an airflow of 2.0–2.5 m/s, and the temperature and humidity were maintained at constant values for 30 min. Based on the data from other experimental studies, the ambient temperature and humidity were set to 42 °C and 65–75%, respectively. Rats were grouped into the following four groups (*n* = 10): Groups: (1) control group, (2) model group, (3) RES^Low^ group, wherein the rats were intraperitoneally injected with 5 mg/kg RES 3 days before and 5 days after modeling (prophylaxis plus treatment lasted for 8 d in total). (4) RES^High^ group, wherein the rats were intraperitoneally injected with 20 mg/kg RES 3 days before and 5 days after modeling (prophylaxis plus treatment lasted for 6 days in total).

In this study, the rat HS model was established based on the method described by Lu et al. [15], with some adjustments. Briefly, after anesthetizing the rats with 35 mg/kg pentobarbital sodium, the femoral artery and vein were cannulated with polyethylene tubing (PE50) cannulae, and the right femoral artery blood pressure was monitored using a small animal physiological monitor (Medlab, Nanjing Calvin Biotechnology Co., Ltd.). Rats were placed in a preheated incubator at 42 ± 0.5 °C and 60 ± 5% relative humidity, with free access to food and water. Rectal temperatures were continuously monitored at 15-min intervals using a rectal thermometer. When the temperature exceeded 42 °C (approximately 60 min in this experiment), it was maintained at 42 °C for approximately 15 min. The moment when mean arterial blood pressure (MAP) began to drop sharply from its peak level was considered to indicate successful HS modeling, at which point the rats were transferred to an environment with a temperature of 24 ± 2 °C and 35 ± 5% humidity for recovery.

Rats were observed for survival within the 5-day post-modeling period, and survival rates were calculated. On day 5, the rats were euthanized using 60% CO_2_, and the peripheral blood was collected through the central abdominal vein to prepare the serum. Lung tissues were fixed and embedded in paraffin for pathological examination. Some lung tissues were used for biochemical index (creatinine [CRE], creatine kinase [CK], LDH, MDA, SOD, GSH-Px, and Fe^2+^) and western blot (SLC7A11, GPX4, HO-1, and Nrf2) analyses.

### Histopathological testing

After paraffin sections of the lung tissue were dewaxed, pathological lung changes were detected using hematoxylin and eosin (HE) staining. Lung tissue sections were subjected to immunohistochemical (IHC) testing, and paraffin sections were dewaxed. Sections were placed in a citrate buffer (pH 6.0) and steamed using a sterilizer at 121 °C for 15 min, following which they were incubated in a 3% hydrogen peroxide solution for 20 min. A blocking solution, containing 1% bovine serum albumin, was then added, and the cells were incubated for 15 min. Primary antibodies against HSP70 (ET1601-11, Huabio), SLC7A11, GPS4, HO-1, and Nrf2 were added, and the cells were incubated overnight at 4 °C. Then, goat anti-rabbit or mouse IgG-horseradish peroxidase secondary antibodies (1:100 dilution) were added and sections were incubated at room temperature for 1 h. After removing the secondary antibody, the sections were re-stained with hematoxylin for 30 s. Common dehydration was followed by clarification with xylene. Neutral gum was used to seal the film. The slides were examined under three different fields using an optical microscope (DM500; Leica) and photographed. The results were quantified using Image-Pro Plus 6.0 (IPP6.0) to analyze the optical density values in three different fields of view.

### Lung tissue biochemical index test

Lung tissues (200 mg) were collected, diluted 1:9 (m/v) in PBS, and homogenized. Centrifugation (3000 × *g*) was performed to obtain the homogenate supernatant for biochemical parameter (LDH, MDA, SOD, GSH-Px, and Fe^2+^) determination. The serum was assayed for the HS markers CK (A032-1-1) and CRE (C011-2-1), these kits were purchased from the Nanjing Jiancheng.

### Effect of Nrf2 silencing on lung injury in RES-protected HS rats

To verify that RES exerts a protective effect against lung injury through the Nrf2 pathway, we injected the rAAV-Nrf2 shRNA recombinant AAV-6 type virus (AAV6 can target lung tissue, provided by Zhejiang Ruyao Biotechnology Co. Ltd, with a viral potency of 5 × 10^13^ v. g/mL) into the tail veins of rats to inhibit the expression of *Nrf2* gene in the lung tissue for reversible experimental studies.

Rats were injected with 250 µL rAAV-Nrf2 shRNA in the tail veins 3 days before HS modeling. The rats were euthanized via CO_2_ asphyxiation on day 5 after HS modeling, and histopathological lung damage was determined. LDH and Fe^2+^ levels in the lung tissues were measured. Lung tissues were subjected to RT-qPCR to detect *Nrf2* expression. The relative protein expression of FTH1, SLC7A11, GPX4, NQO1, HO-1, and Nrf2 was detected using western blotting.

### RT-PCR assay

Total RNA was extracted from rat tissues using the TRIzol reagent. The 1st Strand cDNA Synthesis Kit and gDNA Purge kit were used to reverse transcribe mRNA into cDNA. qPCR was performed using the SYBR qPCR SuperMix Plus (Novoprotein, China) kit and the 7500 Fast Real-Time PCR System (Applied Biosystems; Thermo Fisher Scientific, Inc.). The amplification conditions were as follows kits instructions. β-actin was used as an internal reference gene for normalization, and relative gene expression was calculated using the 2^−△△Ct^ method [16]. The primer sequences were as follows: Rat-*Nrf2*-Forward: 5′-GTC AGC TAC TCC CAG GTT GC-3′, Rat-*Nrf2*-Reverse: 5′-CAG GGC AAG CGA CTG AAA TG-3′; Rat-*β-actin*-Forward: 5′-GTC CAC CCG CGA GTA CAA C-3′, Rat-*β-actin* -Reverse: 5′-TAT CGT CAT CCA TGG CGA ACT GG-3′.

### Statistical analysis

All data from the current study are presented as the mean ± standard deviation of values from triplicate experiments. All results were analyzed using the GraphPad Prism Software (version Prism 8, GraphPad Software, Inc.). Student’s t-test was used for two-group comparisons. *P* < 0.05 was considered to indicate statistical significance.

## Results

### RES treatment alleviates the heat stress-induced reduction in Beas-2B cell viability and oxidative stress injury

**Fig. 1:**
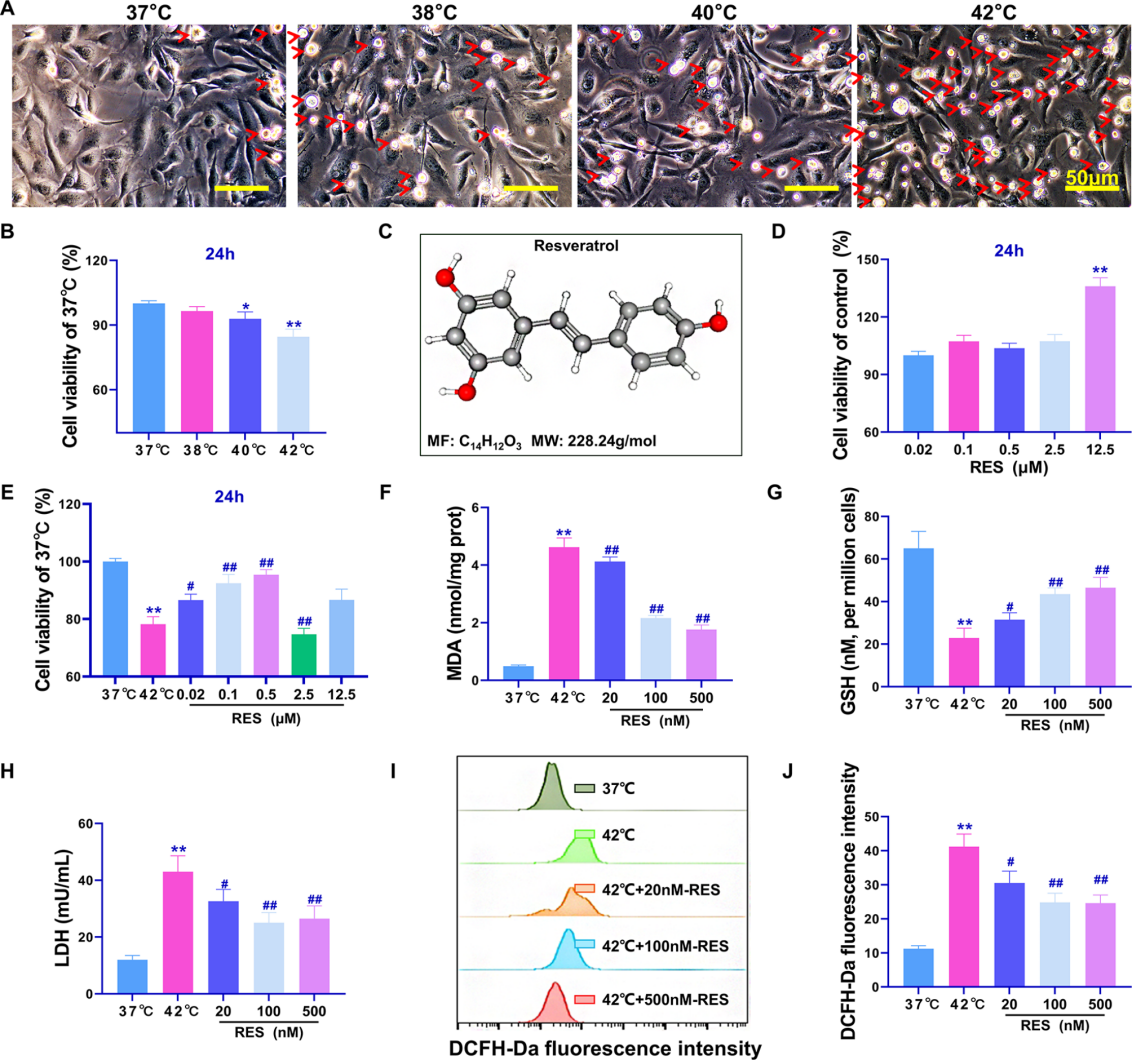
Reduction in Beas-2B cell viability through RES-mediated inhibition of heat stress and oxidative stress induction. (**A**) Phase contrast microscopic observation of the effect of different temperature water bath treatments on cell states. (**B**) MTT assay results regarding the effect of RES on cell viability at 37 °C, 38 °C, 40 °C, and 42 °C; (**C**) RES molecular 3D structure and molecular formula; (**D**) MTT assay results regarding the effects of different doses of RES on the viability of Beas-2B cells. (**E**) MTT assay results regarding the effects of different doses of RES on the viability of Beas-2B cells under heat stress at 42 °C. (**F**-**H**) Biochemical assay for the determination of MDA, GSH, and LDH in cells. (**I**) Flow cytometric detection of fluorescence intensity of the ROS fluorescent probe DCFH-Da. (**J**) FlowJo V10. quantitative statistics of flow cytometry assay results; **P < 0.01, *P < 0.05, compared with the 37 °C group or control group; ##*P* < 0.01, #*P* < 0.05, compared with the 42 °C group, the differences were statistically significant

To the best of our knowledge, this study is the first to demonstrate that heat stress can result in increased Beas-2B cell death. Phase contrast microscopy showed that the number of Beas-2B cells in a suspended circular dead cell state increased with increasing temperatures (Fig. 1A, red clipping heads represent dead cells), the maximum degree of cell death was observed at 42 °C. MTT assay results showed that the most significant reduction in the viability of cells occurred after 2 h of incubation at 42 °C (compared with that in the 37 °C group, *P* < 0.01, Fig. 1B). Figure 1C shows the 3D structural model of RES, whose molecular formula is C_14_H_12_O_3_. The 0–2.5 µM concentrations of RES had no significant effects on cell proliferation and viability, whereas 12.5 µM RES had a significant viability-promoting effect on Beas-2B cells (*P* < 0.01, Fig. 1D). Treatment with RES concentrations of 0.02, 0.1, and 0.5 µM effectively increased the viability of Beas-2B cells under heat stress at 42 °C (Fig. 1E), the results were significantly different (*P* < 0.05) from those in the 42 °C group. When RES concentration exceeded 2.5 µM, cell viability was inhibited (*P* < 0.05). Therefore, 20, 100, and 500 nM working concentrations were used in the subsequent experiments. Assays of oxidative stress indicators showed that RES treatment significantly downregulated MDA and LDH production and upregulated GSH content (*P* < 0.05, Fig. 1F–H). In the ROS accumulation assay, the fluorescence intensity of the ROS fluorescence detection probe DCFH-Da was the highest in the 42 °C group (*P* < 0.01 compared with the 37 °C group), whereas treatment with RES concentrations of 20, 100, and 500 nM significantly inhibited ROS accumulation to some extent (*P* < 0.05 compared with that in the 37 °C group, Fig. 1I-J). In summary, RES attenuated the 42 °C heat stress-induced reduction in Beas-2b cell viability and the oxidative stress-induced damage.

### Effects of RES treatment on heat stress-induced ferroptosis and the Nrf2 pathway in Beas-2B cells

**Fig. 2:**
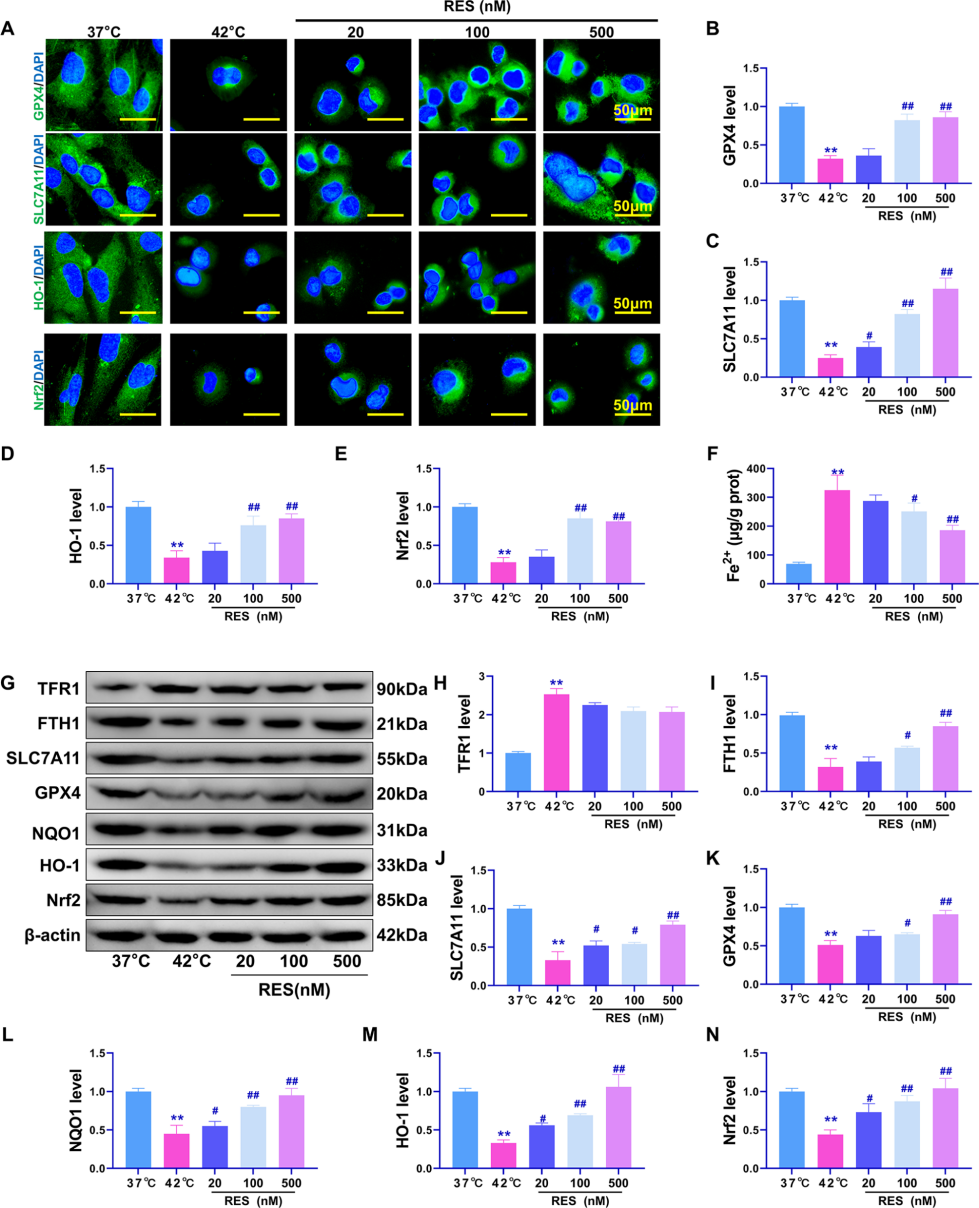
Effect of RES treatment on heat stress-induced ferroptosis. (**A**) Immunofluorescence detection of GPX4, SLC7A11, HO-1, and Nrf2 protein expression and distribution. (**B**-**E**) Quantitative statistical results of immunofluorescent protein intensity. (**F**) Detection of intracellular Fe^2+^ content. (**G**) Western blotting for relative expression levels of the TFR1, FTH1, GPX4, SLC7A11, HO-1, NQO1, and Nrf2 proteins. (**H**–**N**) Quantitative statistical results of the optical density values of western blotted protein bands. **P < 0.01, *P < 0.05, compared with the 37 °C group; ##*P* < 0.01, #*P* < 0.05, compared with the 42 °C group, with statistically significant differences

Iron-dependent accumulation of lipid peroxides usually leads to ferroptosis [7]. Therefore, the expression of Nrf2, HO-1, GPX4, and SLC7A11, which are related to oxidative stress and ferroptosis, was detected using IF (Fig. 2A). Results (Fig. 2B–E) showed that the 42 °C treatment significantly reduced the fluorescence intensity of GPX4, SLC7A11, Nrf2, and HO-1 proteins (*P* < 0.01 compared with that in the 37 °C group). Meanwhile, treatment with 100 and 500 nM RES resulted in significant upregulation of these proteins (*P* < 0.01 compared with that in the 42 °C group). Additionally, treatment with 100 and 500 nM RES significantly reversed the increased intracellular Fe^2+^ content induced by heat stress at 42 °C (*P* < 0.01, Fig. 2F). Western blotting results showed that RES treatment significantly reversed the downregulation of GPX4, SLC7A11, FTH1, and Nrf2 downstream transcriptional regulatory proteins, NQO1 and HO-1, in Beas-2B cells under heat stress (Fig. 2G-N, *P* < 0.05) but had no significant effect on TFR1 protein expression (*P* > 0.05). These results suggest that RES can regulate the Nrf2 pathway to improve the anti-oxidative stress and anti-ferroptotic effects in heat-stressed Beas-2b cells. However, whether Nrf2 is the main pathway involved needs to be further verified using rescue experiments.

### Inhibition of the Nrf2 pathway reverses the resistance of RES to heat stress ferroptosis

**Fig. 3:**
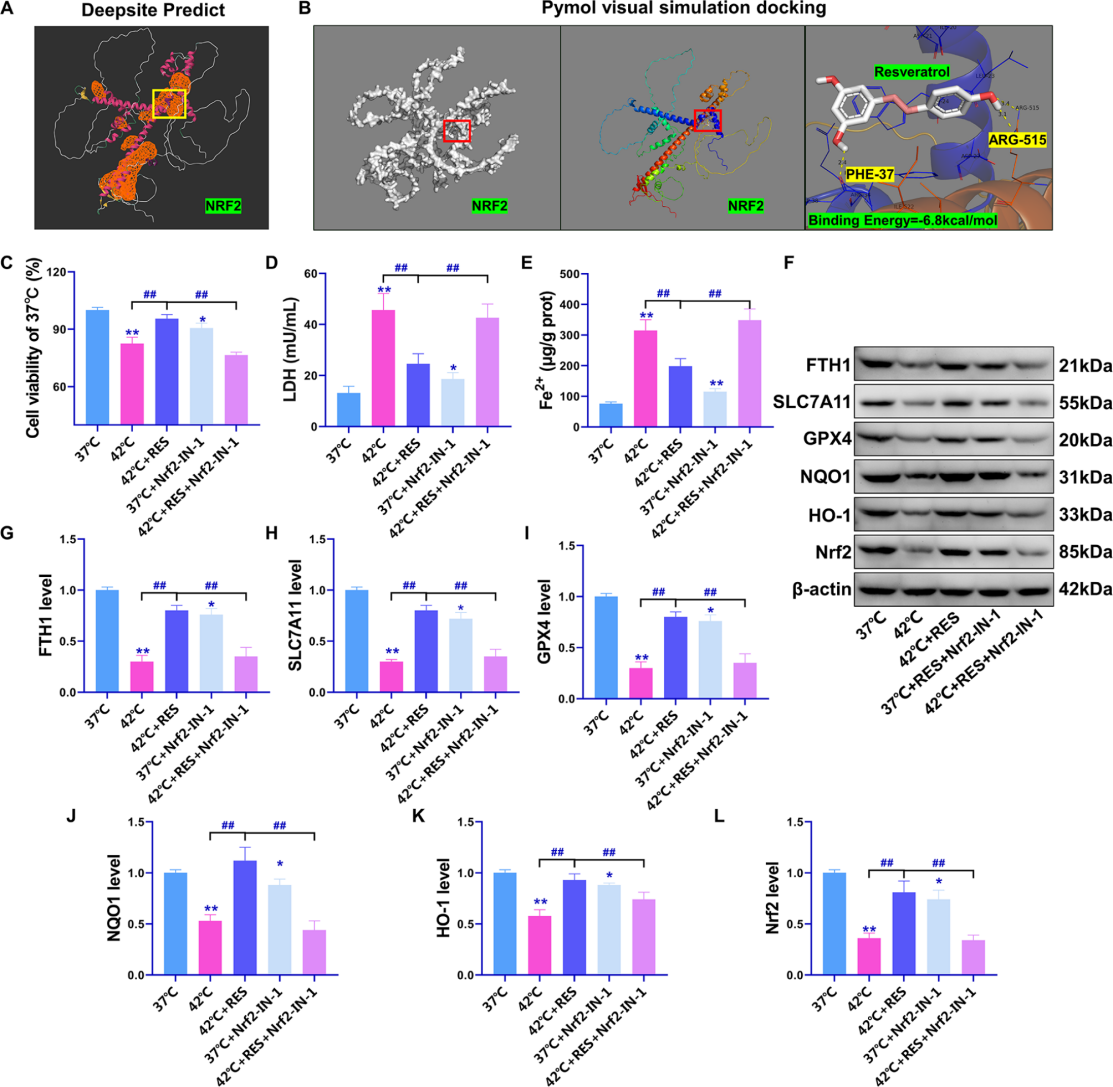
Nrf2-IN-1 significantly reverses the resistance of RES to ferroptosis in Beas-2B cells under heat stress. (**A**) Prediction of Nrf2 protein active pocket sites using Deepsite. (**B**) Visual simulation docking of potential binding relationship between RES and Nrf2 active pocket sites using Pymol. (**C**) MTT assay for cell viability. (**D**) LDH levels, (**E**) intracellular Fe^2+^ content. (**F**) Results of western blotting. (**G**–**L**) Quantitative statistics of protein band optical density values determined using western blotting. **P < 0.01, *P < 0.05, compared with the 37 °C group; ##*P* < 0.01, #*P* < 0.05, the two groups were compared

To investigate whether RES exerts heat stress ferroptosis resistance via the Nrf2 pathway, the Nrf2 inhibitor Nrf2-IN-1 was used for reversibility studies. Before the study began, we predicted direct binding between RES and the Nrf2 protein. The DeepSite tool predicted six active pockets of Nrf2 (orange part in Fig. 3A), among which the yellow box pocket site had the highest predicted binding energy. The coordinate axes were X-axis = -12.26, Y-axis = 13.04, and Z-axis = 7.93. The binding energies of RES and the Nrf2 protein were calculated using the Autodocking software 1.5.6. Results showed that the maximum binding energy of RES was − 6.8 kcal/mol, and there was a potential covalent binding relationship between RES and Nrf2 proteins PHE-37 and ARG-515 (Fig. 3B). Nrf2-IN-1 was found to significantly reverse the effect of RES on the increase in Beas-2B cell viability and reduction of LDH and Fe^2+^ contents under heat stress (compared with those in the 42 °C + RES group, *P* < 0.01, Fig. 3C–E). Western blotting results confirmed that Nrf2-IN-1 significantly inhibited the expression of Nrf2/HO-1/NQO1 pathway-related proteins as well that of the ferroptosis resistance-related proteins GPX4, SLC7A11, and FTH1 (*P* < 0.01 compared with that in the 42 °C + RES group, Fig. 3F–L). In summary, this study confirms that the Nrf2 pathway is one of the main mechanisms through which RES exerts anti-oxidative stress and anti-ferroptotic effects. Till date, this has only been verified at the cellular level, and there are many mechanisms underlying the occurrence and induction of lung injury in thermal radiation disease. To deepen the existing conclusions of this study, we used a rat model of thermal radiation disease to verify the efficacy of RES in providing protection against ferroptosis-induced lung injury through Nrf2.

### Protective effect of RES on HS rats

**Fig. 4:**
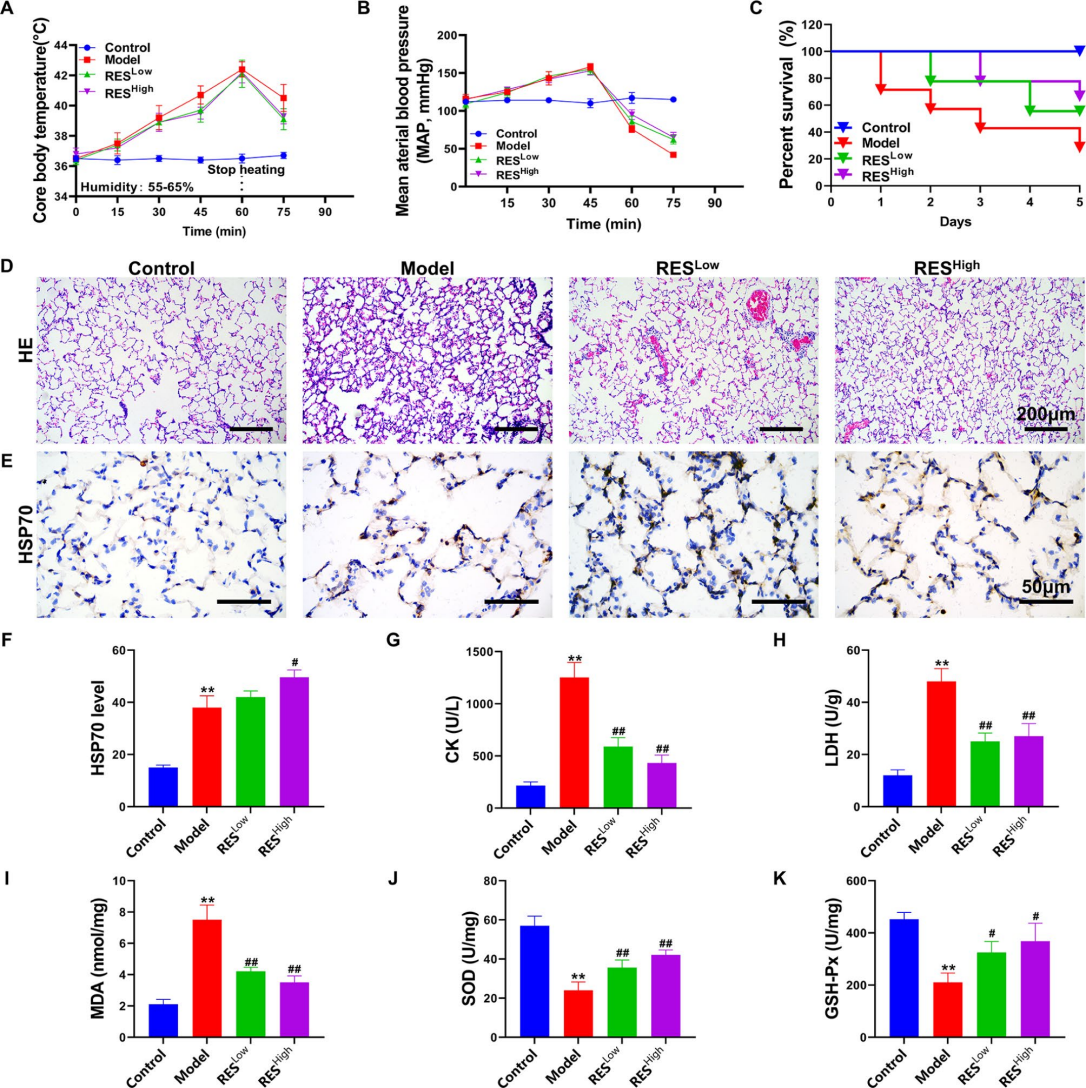
Study of the protective effect of RES on HS rats. (**A**) Anal temperature test results. (**B**) Femoral mean artery blood pressure (MAP) test results. (**C**) Survival rate for each group of rats. (**D**) HE staining of lung tissues. (**E**) HSP70 expression and distribution in lung tissues, as detected via IHC. (**F**) Quantitative statistics of IHC test results. (**G**–**K**) CK, LDH, MDA, SOD, and GSH-Px contents. **P < 0.01, *P < 0.05, compared with the control group; ##*P* < 0.01, #*P* < 0.05, compared with the model group

At a temperature of 42 °C and humidity of 55–65%, the anal temperature of the rats reached approximately 42 °C at 60 min (Fig. 4A). Meanwhile, the arterial blood pressure started to drop abruptly at approximately 45 min at 42 °C (Fig. 4B), suggesting that the rat HS model was successfully established [17]. In the subsequent 5-day survival rate observations, the survival rate of rats in the model group was as low as 30%, whereas the survival rates of the RES^Low^ and RES^High^ groups were 60% and 70%, respectively (Fig. 4C). HE staining revealed interstitial thickening of lung tissue cells, inflammatory cell infiltration with extensive hemorrhagic symptoms, and restricted alveolar expansion in the model group rats (Fig. 4D). Significant stasis of blood was seen in the lung tissue of the RES^Low^ group, but no significant interstitial thickening of the lung was seen. Significant inflammatory cell infiltration was observed around the vessels, and a small amount of stasis was seen in the RES^High^ group, with fewer inflammatory cells and no interstitial thickening of the lung. In addition, RES^High^ induced higher levels of HSP70 protein expression than those in the model group (*P* < 0.05; Fig. 4E-F). The levels of the biochemical indicators CK in the serum and LDH and MDA in tissues were significantly reduced in the RES^Low^ and RES^High^ groups. SOD and GSH-Px levels in the lung tissue were significantly increased compared with those in the model group (*P* < 0.05; Fig. 4G-K). These findings indicate that RES can provide effective protection against acute lung injury and oxidative stress in rats exposed to thermal radiation. However, it is necessary to verify whether ferroptosis was effectively inhibited by RES treatment.

### RES regulates the level of ferroptosis in the lung tissue of HS rats

**Fig. 5:**
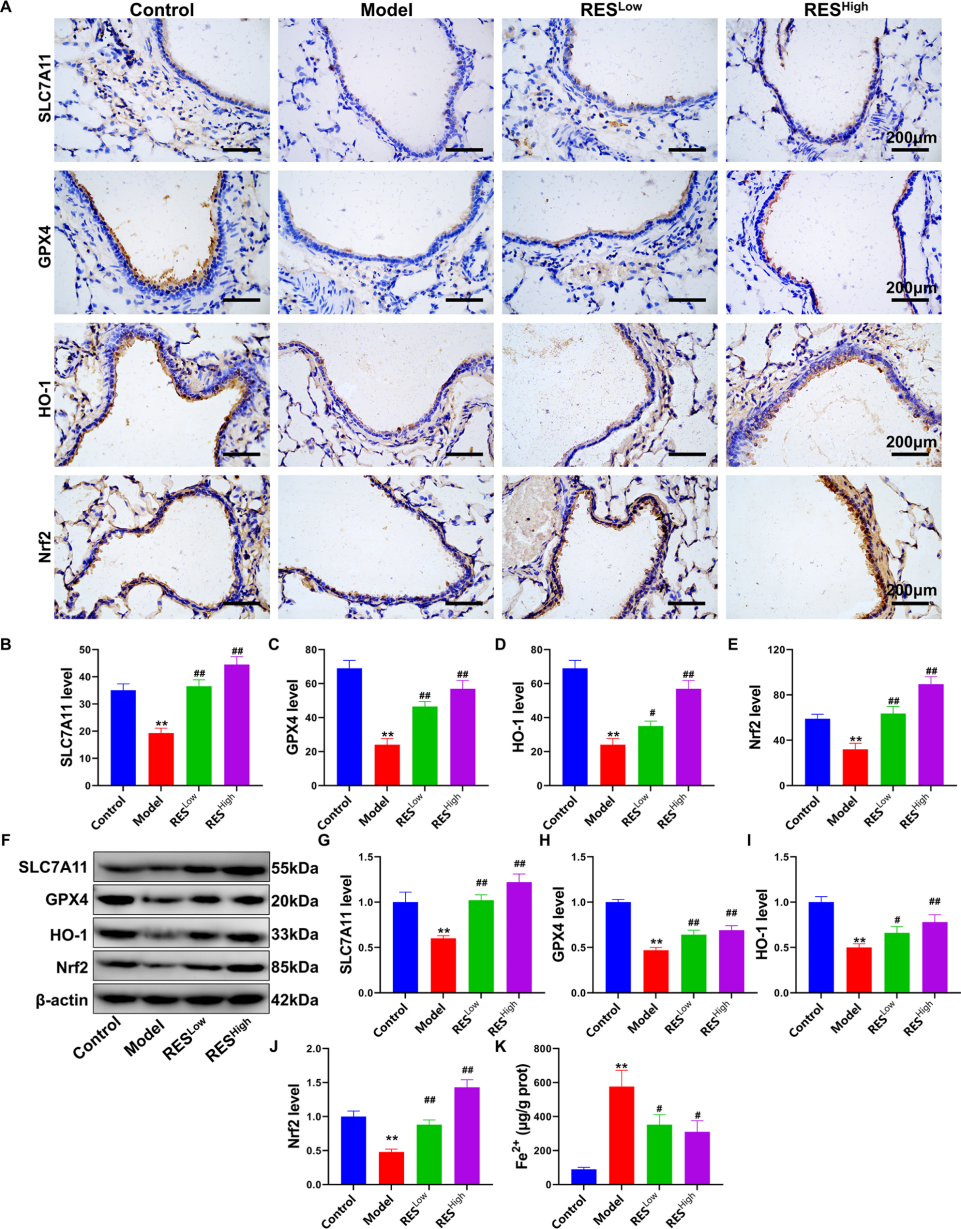
RES regulates the level of ferroptosis in the lung tissues of HS rats. (**A**) Expression and distribution of the SLC7A11, GPX4, HO-1, and Nrf2 proteins in the lung trachea determined using IHC. (**B**–**E**) Quantitative statistics of IHC test results. (**F**) Western blotting for SLC7A11, GPX4, HO-1, and Nrf2 proteins. (**G**–**J**) Quantitative statistics of optical density values of protein bands determined using western blotting. (**K**) Lung tissue Fe^2+^ content. **P < 0.01, *P < 0.05, compared with the control group; ##*P* < 0.01, #*P* < 0.05, compared with the model group

To confirm that RES can induce acute lung injury in heat-induced rats by regulating the ferroptosis pathway, we investigated ferroptosis in lung tissue. First, the expression and distribution of the ferroptosis resistance and oxidative stress protection-related proteins SLC7A11, GPX4, HO-1, and Nrf2 proteins in lung tissues were detected using IHC (Fig. 5A). Results showed that the expression levels of SLC7A11, GPX4, HO-1, and Nrf2 in the lung tracheal epithelial cell sites of HS rats were significantly altered after RES treatment. The adverse effects of HS were significantly reversed by both low and high doses of RES (*P* < 0.05, Fig. 5B–E). This was further confirmed using western blotting (Fig. 5F), which showed that low and high doses of RES significantly upregulated the SLC7A11, GPX4, HO-1, and Nrf2 proteins in lung tissues; the results were significantly different when compared with those of the model group (Fig. 5G–J, *P* < 0.05). And RES also reduced Fe^2+^ contents (Fig. 5K, *P* < 0.05). These findings indicated that RES could inhibit the occurrence of HS-induced ferroptosis in rat lung tissue and that this is related to Nrf2 pathway regulation. Therefore, we further elucidated the underlying mechanisms by conducting rescue experiments using AAV-mediated silencing of Nrf2.

### RES inhibits pulmonary ferroptosis in HS rats via the Nrf2 pathway

**Fig. 6:**
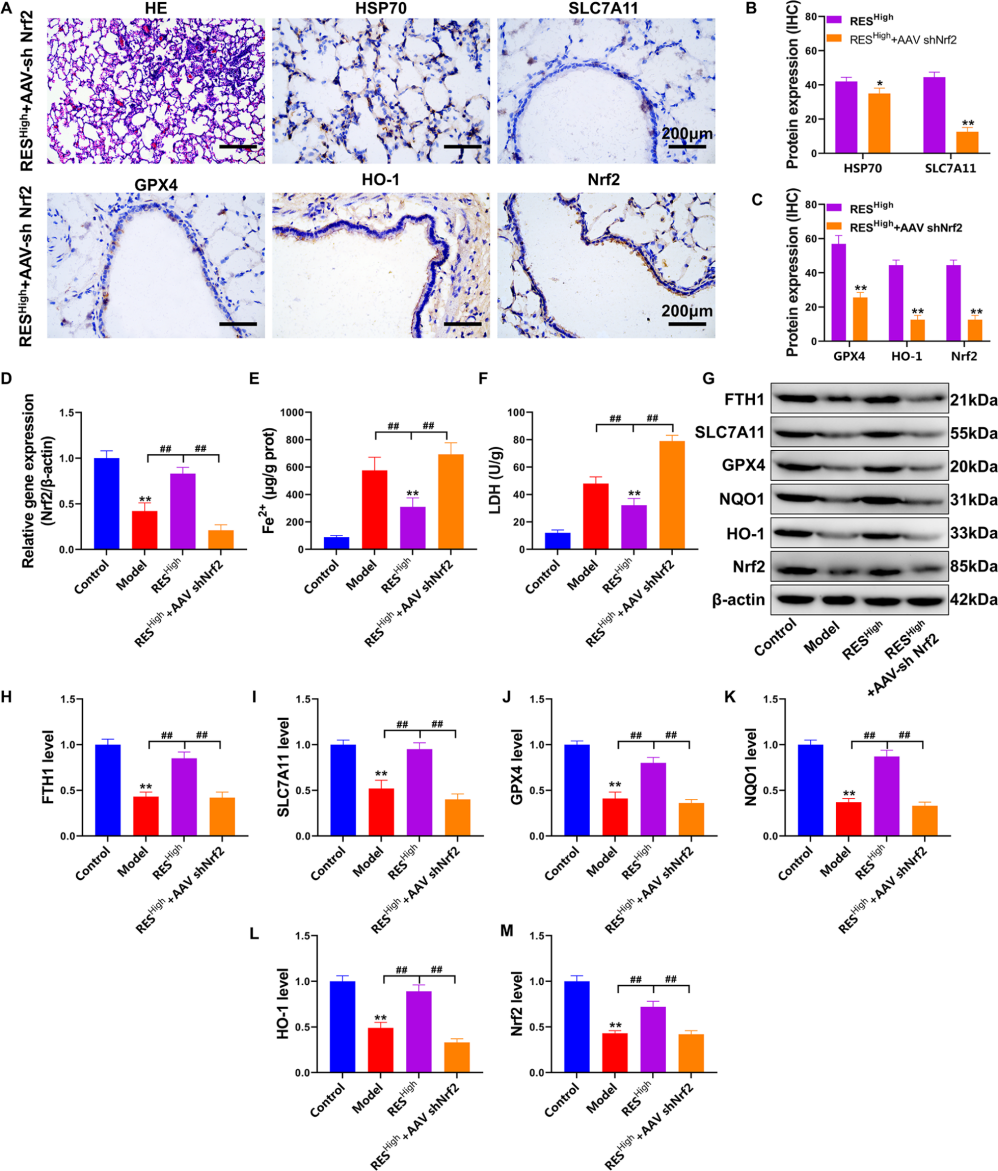
RES rescues pulmonary ferroptosis in HS rats via Nrf2. (**A**) Pathological examination of lung tissue via HE and IHC staining. (**B**-**C**) Quantitative statistics of IHC staining were analyzed and compared with those of the RES^High^ group. (**D**) Detection of *Nrf2* gene expression in lung tissue using RT-PCR. (**E**) Fe^2+^ levels in lung tissue. (**F**) LDH content detection. (**G**) FTH1, SLC7A11, GPX4, NQO1, HO-1, and Nrf2 protein expression levels, as determined using western blotting. (**H**–**M**) Quantification of the optical density values of protein bands determined using western blotting; **P < 0.01, *P < 0.05, compared with the control group; ##*P* < 0.01, #*P* < 0.05, the two groups connected were compared

Nrf2 is a pathway through which RES protects against oxidative stress and injury [18]. To investigate whether RES protects against lung injury via Nrf2 in HS rats, we inhibited pulmonary Nrf2 using tail vein injection of AAV-shNrf2. The results of the pathological study (Fig. 6A) showed that the RES^High^ +AAV shNrf2 group had thickened interstitial lung tissue with massive inflammatory cell infiltration and intravascular stasis (HE results). In addition, the RES^High^ +AAV shNrf2 group exhibited significantly inhibited expression of the HSP70, SLC7A11, GPX4, HO-1, and Nrf2 proteins in the lung tracheal epithelium compared with that in the RES^High^ group (*P* < 0.05; Fig. 6B-C. *Nrf2* gene expression was significantly reduced in HS rats administered RES compared with that in the model group (*P* < 0.01) and then significantly decreased after AAV-shNrf2 injection compared with that in the RES^High^ group (*P* < 0.01, Fig. 6D). In addition, LDH and Fe^2+^ contents in the lung tissue were significantly increased in the RES^High^ +AAV shNrf2 group compared with those in the RES^High^ group (*P* < 0.01; Fig. 6E-F). Western blotting results (Fig. 6G–M) confirmed that the expression of Nrf2/HO-1/NQO1 and the ferroptosis resistance proteins GPX4, SLC7A11, and FTH1 was significantly inhibited in the RES^High^ +AAV shNrf2 group after *Nrf2* gene silencing compared with that in the RES^High^ group (*P* < 0.01). In summary, this study preliminarily confirmed that RES can upregulate the anti-oxidative stress and anti-ferroptotic pathways in Beas-2b cells and rat lungs under HS conditions at the cellular and animal levels and that Nrf2 is the critical target to achieve the above-mentioned protective effects.

## Discussion

In this study, we first induced heat stress in tracheal lung epithelial Beas-2B cells. We confirmed that RES protects against heat stress injury through a mechanism that may be related to oxidative stress injury and ferroptosis inhibition. A recent study showed that RES protects Beas-2B cells from erastin-induced ferroptosis through the Nrf2/Keap1 pathway [19]. The study further demonstrated the protective effect of RES on Beas-2B cells against ferroptosis and that the mechanism of action may involve the classical Nrf2 pathway. However, no studies have been conducted to verify whether RES can protect against ferroptosis via the Nrf2 pathway in heat stress-induced Beas-2B cell injury.

Nrf2 is a transcription factor that regulates intracellular redox homeostasis and intracellular antioxidants that modulate inflammation, senescence, and ROS production. When cells are exposed to oxidative stress, Nrf2 translocates to the nucleus and binds to antioxidant-responsive elements in genes encoding antioxidant enzymes such as NQO1 and HO-1 [18, 20], thereby protecting cells from oxidative stress damage by increasing the expression of these genes.

Regarding the regulatory contribution of Nrf2 to ferroptosis, it has been found that Nrf2 not only inhibits the accumulation of ROS to inhibit ferroptosis by inducing NQO-1 and HO-1 expression [21] but Nrf2 also enhances the transcriptional capacity of SLC7A11 by directly binding to the promoter of the ferroptosis resistance-associated protein SLC7A11 [21]. Although Zhang et al. did not directly demonstrate that GPX4 is transcriptionally regulated by Nrf2 in their chronic obstructive pulmonary disease study, *GPX4* could play a ferroptosis-protective role as a downstream regulatory gene of Nrf2, and the inhibition of *GPX4* expression could block the ferroptosis-protective effect of Nrf2 [22]. Nrf2 also alters iron homeostasis by increasing iron stores and their flux into and out of cells; for example, it induces the expression of the gene that encodes FTH1, which sequesters excess free iron in a protein cage that limits iron redox switching [23]. In the present study, we demonstrated that RES treatment inhibited ROS and Fe^2+^ accumulation in heat-stressed cells and upregulated the Nrf2, NQO-1, HO-1, SLC7A11, GPX4, and FTH1 proteins, which is consistent with the regulation of ferroptosis by Nrf2. These effects were reversed by Nrf2-IN-1, an Nrf2 inhibitor. Another intracellular iron transport-related protein, TFR1, was examined in this study. TFR1 imports iron from the extracellular environment into the cells, contributing to the cellular iron pool required for ferroptosis, and is upregulated to promote ferroptosis [24]. However, no significant regulatory effects of RES on TFR1 were observed in the present study. In a study by Li et al. [25], hypoxic reperfusion induced the downregulation of FTH1 and upregulation of TFR1 in cardiomyocytes, and RES reversed the inhibition of FTH1 protein expression via hypoxic reperfusion. However, no significant modulation of TFR1 protein expression by RES was observed in this study. In cancer research, the Nrf2 antioxidant signaling pathway has been shown to be involved in the protection of hepatocellular carcinoma cells from ferroptosis by regulating the expression of FTH1 but not TFR1 [26]. The results of all the above-mentioned studies are consistent with the phenomena observed in the present study. On the basis of our results, we hypothesized that the protective effect of RES against ferroptosis in Beas-2B cells under heat stress is mediated through the Nrf2 antioxidant pathway. At the animal level, by constructing a targeted Nrf2 gene suppression system in the lungs (using adeno-associated virus AAV6), we confirmed that RES activated the Nrf2 pathway and alleviated lung injury, resulting in anti-oxidative stress and anti-ferroptotic effects.

In addition to detecting pathological damage to the lungs, we examined pathological damage to the kidney tissue (Supplementary Fig. 1). While RES exerted adequate protective effects on the lung tissue, no significant effect was observed in renal tissues, particularly against the HS-induced renal tubular damage. CRE is commonly used as an indicator of HS injury, and abnormal elevations are considered indicative of renal failure [27]. However, no significant modulatory effect of RES on CRE was observed in the present study (the difference was not statistically significant when compared with the results of the model group), which is consistent with the degree of injury in the pathology. Accordingly, we suggest that in a follow-up experimental study, a combination treatment with RES and drugs related to renal injury treatment may result in more significant protective effects against HS.

This study had some limitations: First, a single thermal radiation disease model was used in this study. Currently, there are usually two kinds of thermal radiation disease models; one is a classic heat stroke model, wherein excessive heat exposure causes the body core temperature to rise to more than 42 °C; this model was used in the current study. The other is exertional HS, which is caused by an imbalance between the human body’s heat production and heat dissipation caused by intense physical activity [28]. Clinical differences exist between the two febrile diseases, such as the time of disease occurrence, rate of disease progression, and inducement [29]. Therefore, we need to combine EHS experiments to further expand the evidence for the efficacy of RES in treating HS diseases. Second, this study only examined the protective effects on the lung and kidney tissues. No significant protective effect of RES was observed in the kidneys, suggesting that when RES is used for HS treatment, it is also necessary to combine renoprotective drugs for collaborative treatment, as kidney injury is crucial in HS disease [30]. In addition, there is a lack evidence for the protective effects of RES on other organs with thermal eject-related dysfunction, such as the liver, intestines, and heart, which are essential for a comprehensive evaluation of the HS efficacy of RES. Third, the sample size in the animal experiments was small. Studies on the long-term impact of RES on the prognosis of patients with HS are lacking because the incidence of cardiovascular diseases and chronic nervous system sequelae in survivors of HS has significantly increased. Meanwhile, RES has been proven to reduce the incidence of cardiovascular and neurological diseases [31]. In addition, RES is convenient and exhibits preliminary clinical effects regarding protection against cardiovascular diseases, which is crucial in improving the quality of life of HS survivors and has substantial medicinal value [32]. Therefore, the long-term protective effect of RES on HS and the more far-reaching prognostic mechanism of HS warrants further study.

## Conclusion

In summary, this study confirmed the protective effects of RES in an HS model using Beas-2B cells and rats. The results suggest that RES induces oxidative stress and ferroptosis resistance via the Nrf2 antioxidant pathway. Although adequate lung injury protection was observed in this study, the protective effect of RES in other organs, especially the kidneys, was weak. Our study provides an experimental basis for treating the lung injury caused by heat stroke and offers additional medicinal options for the clinical treatment of heat stroke. However, to comprehensively treat HS, it may be necessary to adopt a combination of drugs to obtain more significant benefits, including protection against acute kidney injury. Adding drugs to HS therapies has far-reaching significance in improving the overall patient outcomes.

## Electronic supplementary material

Below is the link to the electronic supplementary material.

Supplementary Material 1

Supplementary Material 2

## Data Availability

No, I don’t have any research data outside the submitted manuscript file.

## Abbreviations

BSA: Bovine serum albumin
CK: Creatine kinase
CRE: Creatinine
HS: Heat stroke
GSH: Glutathione
GSH-Px: Glutathione peroxidase
HE: Hematoxylin and eosin
HO-1: Heme oxygenase-1
HRP: Horseradish peroxidase
IHC: Immunohistochemical
LDH: Lactate dehydrogenase
MAP: Mean arterial blood pressure
MDA: Malondialdehyde
Nrf2: Nuclear factor-2 associated factor
RES: Resveratrol
ROS: Reactive oxygen species
