# Supplementary material for: Resveratrol inhibits ferroptosis in the lung tissues of heat stroke-induced rats via the Nrf2 pathway
Source: BMC Pharmacol Toxicol. 2024 Nov 19;25:88. doi: 10.1186/s40360-024-00810-1 (PMC11577854; doi:10.1186/s40360-024-00810-1)
Supplement: Supplementary file 1 — Supplementary Material 1 [file 40360_2024_810_MOESM1_ESM.doc]

Figure 2


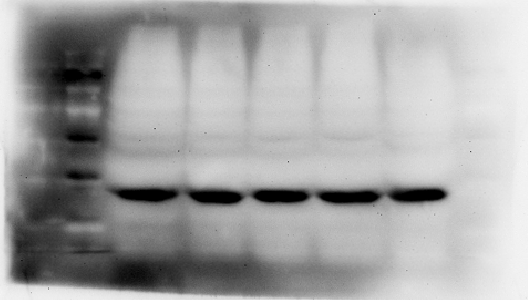
beta-actin


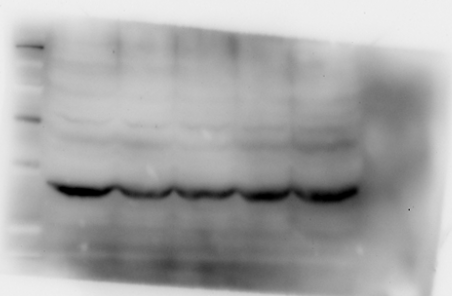
GPX4


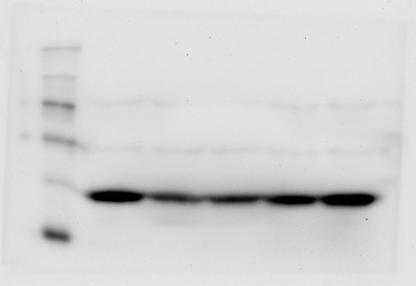
HO-1


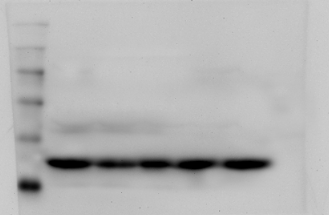
NQO1


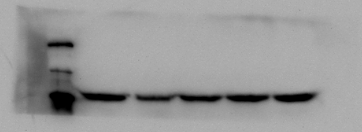
NRF2


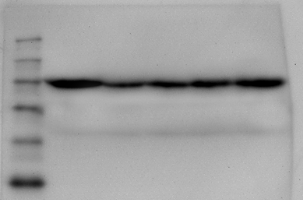
SLC7A11


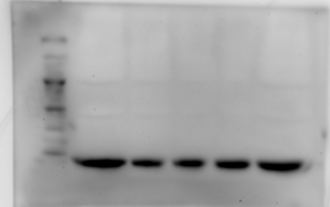
TFH1


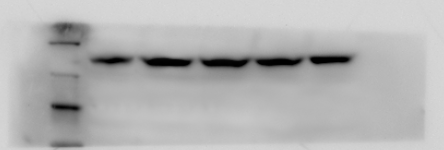
TFR1

Figure 3


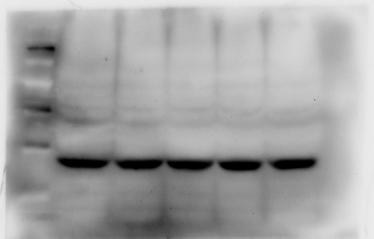
beta-actin


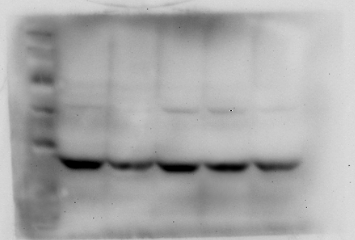
FTH1


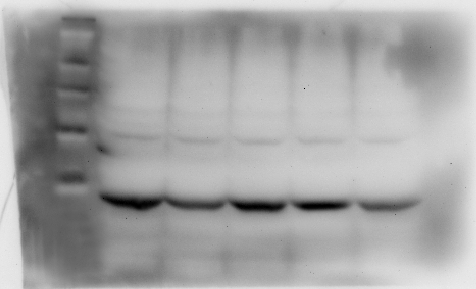
GPX4


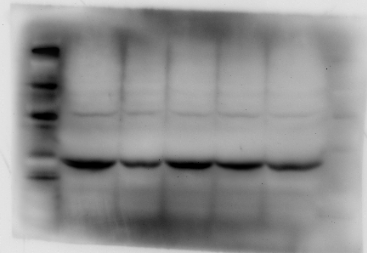
HO1


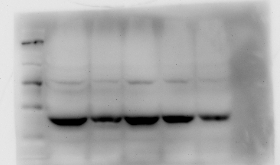
NQO1


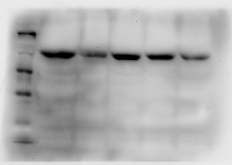
NRF2


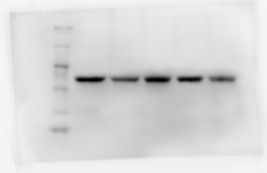
SLC7A11

Figure 5


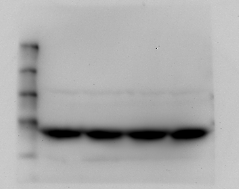
beta-actin


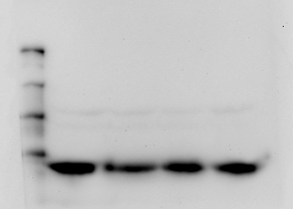
GPX4


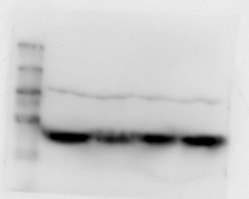
HO1


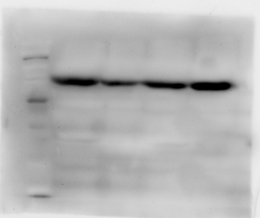
NRF2


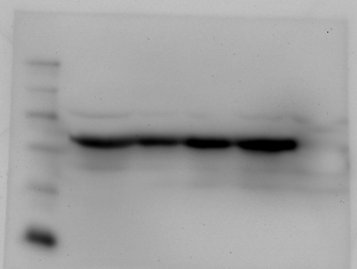
SLC7A11

Figure 6


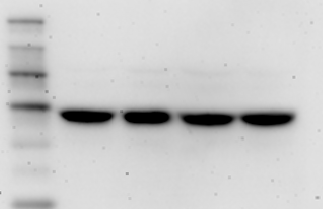
beta-actin


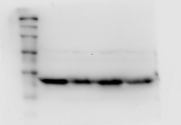
FTH1


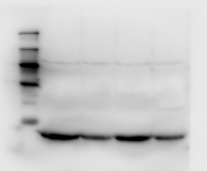
GPX4


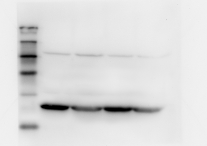
HO1


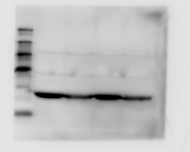
NQO1


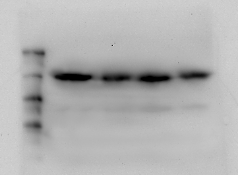
NRF2


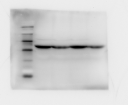
SLC7A11
