# Supplementary figures and images for: Resveratrol inhibits ferroptosis in the lung tissues of heat stroke-induced rats via the Nrf2 pathway
Source: BMC Pharmacol Toxicol. 2024 Nov 19;25:88. doi: 10.1186/s40360-024-00810-1 (PMC11577854; doi:10.1186/s40360-024-00810-1)

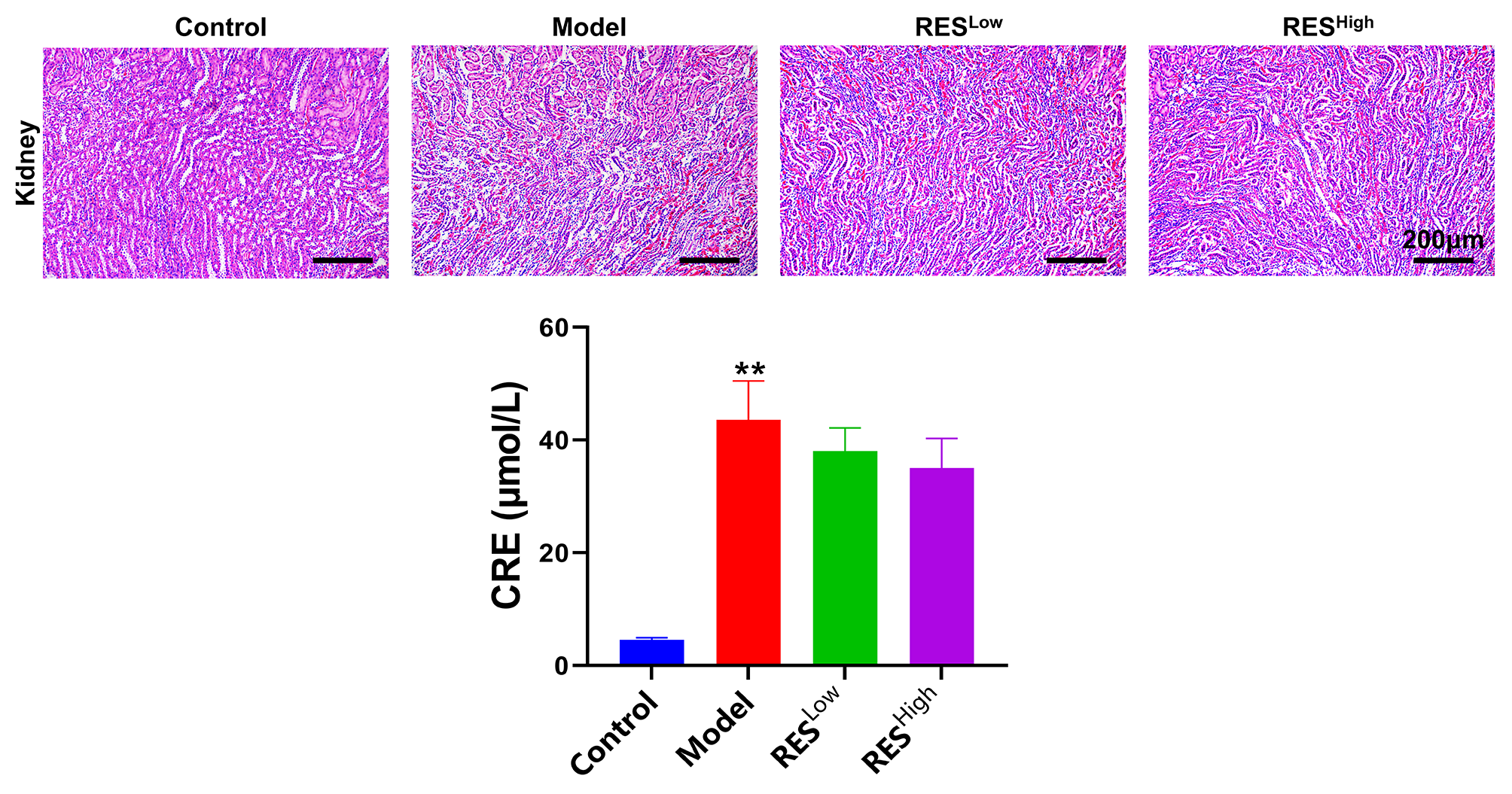

Supplement: Supplementary file 2 — Supplementary Material 2 [file 40360_2024_810_MOESM2_ESM.tif]
